# Applicability of tools to identify potentially inappropriate prescribing in elderly during medication review: Comparison of STOPP/START version 2, Beers 2019, EU(7)-PIM list, PRISCUS list, and Amsterdam tool—A pilot study

**DOI:** 10.1371/journal.pone.0275456

**Published:** 2022-09-29

**Authors:** Agnieszka Lisowska, Edyta Czepielewska, Martyna Rydz, Anna Dworakowska, Magdalena Makarewicz-Wujec, Małgorzata Kozłowska-Wojciechowska

**Affiliations:** Faculty of Pharmacy, Department of Clinical Pharmacy and Pharmaceutical Care, Medical University of Warsaw, Warsaw, Poland; University Campus Bio-Medico di Roma, ITALY

## Abstract

Potentially inappropriate prescribing (PIP) is one of the major risk factors of adverse drug events in elderly patients. Pharmacotherapy assessment criteria may help reduce the instances of PIP among geriatric patients. This study aimed to verify the applicability of selected tools designed to assess prescribing appropriateness in elderly and to identify PIP in the study population. Based on pharmacist-led medication reviews that were performed among patients attending senior day-care centers based in Poland, aged 65 years and over, the following tools were applied for assessing the appropriateness of pharmacotherapy: PILA (patient-in-focus listing approach): STOPP/START v.2 and Amsterdam tool, DOLA (drug-oriented listing approach): PRISCUS list, and DOLA+: Beers criteria v.2019 and the EU(7)-PIM list–the criteria oriented on medications requiring indications. Fifty patients participated in the study. The prevalence of prescribing issues in the study population was very high and ranged from 28% to 100%, depending on the criteria applied. The highest number of PIP cases was identified based on the PILA criteria: STOPP/START v.2 (171, a mean of 3.4 PIP cases per patient), and the Amsterdam criteria (124, a mean of 2.5 PIP cases per patient). The lack of protective vaccinations against pneumococci identified using the START criterion was found to be the most common PIP (identified in 96% of the patients). Proton-pump inhibitors (PPIs) were identified as the most problematic group of medications. The STOPP, EU(7)-PIM and Beers criteria revealed cases of inappropriate prolonged PPI use, whereas the Amsterdam tool identified cases where PPIs should have been prescribed but were not. The highest number of PIP cases in the study population were identified with the PILA tools, and on this basis the most comprehensive assessment of pharmacotherapy appropriateness in geriatric patients was conducted. Further studies should be designed, covering a larger group of patients across different healthcare settings (inpatient and outpatient), with access to comprehensive patient data.

## Introduction

Worldwide, people aged 65 years or over is the fastest growing age group in the general population. It is predicted that by 2050, every sixth person in the world, every fourth person in Europe and every third person in Poland will have reached this age [1,2]. With the high proportion of elderly in the general population, more emphasis needs to be given to improvements in geriatric care.

The specific characteristics of the geriatric population may render prescribing medications more difficult. The ageing process involves progressive physiological deterioration and has a negative effect on the homeostatic mechanisms. Combined with the age-related changes in pharmacodynamics and pharmacokinetics, these factors increase the risk of adverse drug events (ADEs). Moreover, the use of multiple medications, common in the elderly, makes the choice of appropriate pharmacotherapy even more complex in this patient population.

Potentially inappropriate prescribing (PIP) to elderly is defined as pharmacotherapy that fails to follow the accepted medical standards. This can include potentially inappropriate medications–PIMs, in which case the drug-carrying risk outweighs the potential benefits, and potentially prescription omissions–PPOs, which means not prescribing a beneficial medicine although it is not contraindicated. Inappropriate pharmacotherapy was found to be very frequent in the geriatric population. Depending on the applied criteria used to determine inappropriate prescribing, it affects up to 98% of geriatric patients [3]. Additionally, inappropriate prescribing was shown to be one of the major risk factors of ADEs in the elderly [4].

Medication reviews conducted by pharmacists, in any healthcare setting, may constitute a starting point for assessing the appropriateness of prescribing to elderly, and consequently, for improving the quality of pharmacotherapy in these patients [5,6]. The Pharmaceutical Care Network Europe (PCNE) names three principal types of medication reviews (MRs): type 1 –simple, type 2 –intermediate, and type 3 –advanced [7]. Each subsequent type of MRs requires more comprehensive patient data and more detailed information on the prescribed pharmacotherapy, and therefore it may provide more insights about irregularities in pharmacotherapy. The PCNE classification is based on the assumption that pharmacists have access to information about all medications that a patient is currently taking or may have recently stopped taking. Unfortunately, there is no single integrated system in place in Poland that would collect patient information to make it available to pharmacists and to provide all the data essential for a medication review. As a result, pharmacists have to rely on information obtained directly from patients.

There are tools identifying potentially inappropriate prescribing that can be used to provide more effective assessment of the prescribing patterns. The number of tools that can be used in prescribing assessments is truly impressive. In a recent systematic review, Pazan et al. identified 76 tools designed for assessing the appropriateness of prescribing [8]. An ideal tool does not exist, and each of the available measures has its strong and weak points; the choice of the tool may depend on its intended purpose (i.e. daily practice or advanced medication reviews), and the data availability [9]. Moreover, their applicability in other countries may be limited due to the origins of specific criteria, accounting for national recommendations, differences in medication availability, and country-specific prescribing practices [10].

So far, the tools for assessing the appropriateness of prescribing in elderly have been classified as implicit–based on the clinical assessment of individual patients, and explicit–based on pre-defined criteria, most often centered around specific medications and/or diseases [11]. However, this arbitrary classification may be confusing as some of the explicit criteria require detailed patient data. In a new approach, Pazan et al. specified three categories of these tools: PILA–patient-in-focus listing approach; requiring in-depth knowledge about patients, providing structured answers, both positive and negative, to questions regarding the patient’s underlying conditions; DOLA–drug-oriented listing approach, in which detailed knowledge about patients is not mandatory, providing a set of predominantly negative or only negative suggestions; DOLA+–it is different from DOLA only in the fact that it contains recommendations associated with specific indications, therefore it requires knowledge of the indications [8].

There have been few studies on the prevalence of potentially inappropriate prescribing in the geriatric population in Eastern Europe [12–16]. Moreover, there is a lack of easily applicable tools specific for this region that may be used to identify PIP. Therefore, this study aimed to verify the applicability of selected tools for assessing the appropriateness of geriatric pharmacotherapy in Poland in conditions of limited access to patient data, and to identify potentially inappropriate prescribing in the study population.

## Materials and methods

The study population consisted of patients attending senior day-care centers based in Poland (3 senior day-care centers located in different districts of Warsaw), aged 65 years and over, receiving at least three medications per day. Patients under all-day care provided by third parties were excluded. Fifty patients who met the inclusion criteria were enrolled in the study (17-17-16 patients per senior day-care center). Sociodemographic data, health behaviors, medical history, vaccination history, as well information on medications (Rx and OTC) and dietary supplements, including their form and dosage, were collected during direct interviews with the patients. The patients were also asked about the reasons why they were prescribed each medication, whether they adhered to the therapy prescribed, and if they had concerns associated with the pharmacotherapy. The interview form is included in S1 Appendix.

The analyzed medication reviews were conducted from January to May 2018 by pharmacists from the Department of Clinical Pharmacy and Pharmaceutical Care, Medical University of Warsaw. Before interviews with the patients, each patient was informed about the purpose of this research, and signed an informed consent form to participate in the study. To guarantee consistent assessment of PIP, the results of the medication reviews were discussed in a group of pharmacists participating in the study.

Five tools were used to assess the appropriateness of prescribing: one drug-oriented tool (DOLA)–PRISCUS list; two drug-oriented tools that required indications (DOLA+): Beers criteria v. 2019 and the EU(7)-PIM list, and two patient-oriented tools (PILA): the Amsterdam tool and STOPP/START criteria v.2 [17–21]. Due to their specific structure, the individual tools required different approaches. The pharmacotherapy of patients was analyzed with the use of PRISCUS and EU(7)-PIM lists according to the active substances listed. The STOPP/START criteria are broken down into organ systems, and therefore the screening for medication errors was conducted based on complaints reported by patients as well as indications for the use of medication groups prescribed. The Amsterdam tool is based on disease classification; if a patient did not report a condition listed in the criteria, the analyst proceeded to the next section. The Beers criteria consist of several sections: medications to be avoided in the elderly (part 1), medications to be avoided in combination with specific diseases or syndromes (part 2), medications to be used with caution (part 3), and potentially clinically important drug interactions to be avoided (part 4). The first and the third section were analyzed by screening for active substances prescribed. The reported symptoms were analyzed in the second section, and the fourth section was screened for groups of medications prescribed.

Statistical analysis were performed using Microsoft Excel 365. The characteristics of the study population and the number of PIP cases per patient are expressed as means ± standard deviation (SD).

The study was approved by the Ethics Committee at the Medical University of Warsaw.

## Results

The study group consisted of 50 patients, including 39 women (78%). The mean age was 77.1±7.1 years. The patients were taking a total of 474 medications. Each individual was taking a mean of 9.5±4.2 medications (3 to 21), of which on average 6.1±3.0 were prescription-only drugs. The most common conditions reported by the study participants were cardiovascular diseases, motor system diseases, metabolic diseases, gastrointestinal diseases, and sleeping disorders. Table 1 shows the baseline characteristics of the study population.

**Table 1:** Demographic and clinical characteristics of respondents.

Characteristics	Number (%)
Age (years)	
65–74	19 (38)
75–84	22 (44)
≥85	9 (18)
Gender	
Female	39 (78)
Number of reported diseases	
1–4	26 (52)
5–8	23 (46)
≥9	1 (2)
The most commonly reported diseases	
Hypertension	23 (46)
Joint disorders	14 (28)
Dyslipidemia	13 (26)
Osteoporosis	11 (22)
Diabetes	11 (22)
Sleeping disorders	10 (20)
Hypothyroidism	7 (14)
Peptic ulcer	6 (12)
GERD*	5 (10)
Number of medications	
3–4	4 (8)
5–9	25 (50)
≥10	21 (42)

*GERD—gastroesophageal reflux disease.

The prevalence of drug-related problems (DRPs) in the study population, i.e. the percentage of patients in whom at least one PIP was detected, was very high and ranged from 28% to 100%, depending on the criteria applied. The highest number of PIP cases was detected with the patient-oriented criteria (PILA), i.e. STOPP/START, which identified DRPs in each patient, and the Amsterdam tool that detected PIP in 96% of the patients. The lowest (28%) prevalence of PIP in the patients was found using the PRISCUS list. Apart from the PRISCUS list, all of the applied criteria detected PIP in more than 70% of the patients. The number of patients with PIP, the mean number per patient and the total number of PIP cases identified by the different criteria are presented in Table 2.

**Table 2:** The number (%) of patients with PIP, the mean number of PIP cases per patient ± SD and the total number of PIP cases identified by the different criteria.

Criteria	Number (%) of patients with PIP						Mean PIP/ patient±SD	Total PIP
	1	2	3	4	≥5	At least 1 (PIP)		
STOPP v.2	19 (38)	15 (30)	7 (14)	2 (4)	1 (2)	44 (88)	1.7±1.1	83
START v.2	19 (38)	22 (44)	7 (14)	1 (2)	0 (0)	49 (98)	1.8±0.8	88
STOPP/ START v.2	0 (0)	13 (26)	17 (34)	11 (22)	9 (18)	50 (100)	3.4±1.3	171
Amsterdam tool	12 (24)	14 (28)	12 (24)	5 (10)	5 (10)	48 (96)	2.5±1.5	124
Beers 2019	16 (32)	14 (28)	6 (12)	2 (4)	1 (2)	39 (78)	1.5±1.2	75
EU(7)-PIM	21 (42)	12 (24)	3 (6)	0 (0)	0 (0)	36 (72)	1.1±0.9	54
PRISCUS	9 (18)	3 (6)	1 (2)	1 (2)	0 (0)	14 (28)	0.4±0.9	22

Drug- and/or disease-oriented tools (DOLA) were the easiest to use, but detected the lowest number of PIP cases in the fewest patients. The PRISCUS list identified 3.5 times fewer cases of PIP than patient-oriented tools (PILA), and on average 2.5 times fewer cases of PIP than the tools that accounted for the indications (DOLA+). The DOLA+ tools identified PIP in nearly three-fourths of patients.

In the study population, the lack of appropriate prophylaxis with protective vaccinations against pneumococci was found to be the most common PIP (in 96% of patients) identified according to the START criterion. The lack of or unclear indications for at least one medication prescribed was the second most prevalent PIP (in 82% of patients). This problem was detected by the STOPP and the Amsterdam criteria. Multivitamin formulations and formulations containing magnesium were the most common medications taken despite insufficient indications. The Beers criterion identified prescribing that increased the risk of SIADH (syndrome of inappropriate antidiuretic hormone secretion) or hyponatremia in more than half of the patients (52%). The STOPP, EU(7)-PIM and Beers criteria revealed excessive use of PPIs (proton-pump inhibitors); the STOPP and EU(7)-PIM criteria–in 32% of patients (despite patient’s clinical status and individual indications), the Beers criterion–in 22% of patients, on the presumption that PPI use > eight weeks was PIM except for high-risk patients. The Beers criterion also detected excessive aspirin use in primary prevention (in 22% of the patients). Conversely, the Amsterdam tool identified the absence of gastric acid protection in 18% of patients using anticoagulants and in 14% of patients taking NSAIDs. The Amsterdam tool brings attention to the fear for adverse drug events, which occurred in 20% of patients. The STOPP, Beers and PRISCUS criteria identified the use of hypnotic Z-drugs, which increase the risk of falls in elderly. The most prevalent PIP cases for each set of criteria are presented in S1 Table.

## Discussion

To the best of our knowledge, the most recent versions of the five criteria: the STOPP/START v.2, the Amsterdam tool, the EU(7)-PIM list, the PRISCUS list, and the Beers criteria (2019) have not yet been applied in parallel to one patient collective.

In the geriatric population, most attention is drawn to PIMs prescribed to patients, whereas the problem of underprescribing is often neglected. This is also reflected in the fact that only a fraction of appropriateness assessment criteria designed for elderly patients take this aspect into account. However, according to the studies conducted in Europe, the underprescribing of drugs with proven benefits in geriatric patients is widespread and may affect more than half of these patients [22,23]. PPOs, which turned out to be the most common problem in the analyzed population, were identified only by the PILA tools (START). Absence of vaccinations against pneumococci was detected in almost all of the study patients (96%).

The lack of or unclear indications for at least one medication was the second most prevalent PIP. Multivitamin formulations and formulations containing magnesium were the most common medications taken despite the lack of concrete indications. The commonality of this problem can be attributed to the specificity of the geriatric population in Poland. As demonstrated by Cybulski et al., nearly 70% of people aged ≥60 years in Poland take vitamins and dietary supplements, by far more than any other populations analyzed [24]. This PIP was identified using the PILA tools (Amsterdam tool and STOPP/START), and the STOPP/START criteria applied separately, including DOLA+ (STOPP). This can be explained by the fact that, according to Pazan’s classification, the STOPP criteria applied separately are classified as DOLA+, whereas the START criteria are classified as PILA [8]. It is worth noting that, when the STOPP and the START criteria were applied separately in the analysis, the Amsterdam tool identified the highest number of PIP cases among the highest number of patients (a mean of 2.5 PIP cases per patient). However, the highest prevalence of PIP was still identified using the START criteria (98%). The prevalence of PIP identified with the Amsterdam tool equaled 96%, which corresponds to the results of the study by Ahmad et al., in which the Amsterdam tool was applied to analyze the appropriateness of pharmacotherapy in 340 geriatric patients discharged from hospitals in the Amsterdam area [25]. The high rates of PIP in the study populations may be attributed to the fact that the Amsterdam tool detects a very wide range of drug-related problems. It is the only tool that accounts for DRPs from the patient’s perspective and tackles this issue in a dedicated section. This allows for the detection of problems that may affect patient adherence and ultimately lead to treatment discontinuation.

In the study population, PPIs proved to be the most problematic group of medications. According to the latest criteria, this group of medications is increasingly often discussed in the context of possible PIP [26,27]. The STOPP, EU(7)-PIM and Beers criteria can be used to recognize prolonged use of PPIs as inappropriate. Specifically, prolonged (>8 weeks) use of PPIs in elderly may be associated with an increased risk of fractures, bone loss, and *Clostridium difficile* infection. However, the Amsterdam tool enables to identify cases where PPIs should have been prescribed, but were not, such as with the use of anticoagulants or NSAIDs.

The drug-oriented and/or disease-oriented tools (DOLA and DOLA+) identified fewer PIP cases than the patient-oriented criteria (PILA), and although they seem easier to use in daily practice, they should be regularly adapted to new developments and requirements of the pharmaceutical market and the availability of products. Hence, it seems that the PILA criteria (STOPP/START and Amsterdam tool), which are more easily adapted to the realities of different markets, are a better option for healthcare professionals involved in the care of elderly patients, particularly that, as shown in this study, they can detect more instances of PIP. In Poland, due to the shortage of geriatricians, elderly patients are usually managed by either general practitioners and medical specialists [28]. This is a widespread practice in the health care systems of Central and Eastern Europe. As a result, the attending physicians tend to focus on individual conditions rather than optimize treatment to multiple comorbidities, which translates into an increased risk of PIP. The PILA criteria, with a holistic approach to patient treatment, especially when introduced into an electronic system to support the decision-making by prescribers, could reduce the risk of PIP. These criteria could also support pharmacists in more easily identifying omissions or potential prescribing errors before dispensing medications to patients.

This study also proved that pharmacists in Poland may identify many drug problems using the appropriate tools to increase the safety and effectiveness of pharmacotherapy.

### Limitations of the study

This is a pilot study. The limited size of the study population, the cross-sectional nature, and the lack of cross-referencing of the interview data with clinical data may have affected the results of the study. In order to confirm the results obtained, it is necessary to plan further studies involving a larger group of patients, in both inpatient and outpatient settings, with access to comprehensive clinical data.

## Conclusions

The prevalence of prescribing problems in the study population was very high and ranged from 28% to 100%, depending on the applied criteria. Despite limited access to data, the PILA tools (STOPP/START v.2 and Amsterdam tool) identified the highest number of PIP cases in the study population. These patient-oriented tools allowed for the most comprehensive assessment of pharmacotherapy appropriateness in geriatric patients. The lack of appropriate prevention with protective vaccinations against pneumococci was the most common PIP (in 96% of patients) identified with the START criterion. PPIs proved to be the most problematic group of medications. The STOPP, EU(7)-PIM and Beers criteria can detect inappropriate prescribing of long-term therapy with PPIs, whereas the Amsterdam tool identifies cases where PPIs should have been prescribed but were not. Research should continue to compare PIP criteria across a larger patient population, in both inpatient and outpatient settings, with access to comprehensive clinical data.

## Supporting information

S1 Table The most prevalent PIP cases for each set of criteria. (PDF)

S1 Appendix Interview form. (PDF)

S2 Appendix Data underlying described results. (PDF)
