# Supplementary material for: Applicability of tools to identify potentially inappropriate prescribing in elderly during medication review: Comparison of STOPP/START version 2, Beers 2019, EU(7)-PIM list, PRISCUS list, and Amsterdam tool—A pilot study
Source: PLoS One. 2022 Sep 29;17(9):e0275456. doi: 10.1371/journal.pone.0275456 (PMC9521918; doi:10.1371/journal.pone.0275456)
Supplement: S1 Table — (PDF) [file pone.0275456.s001.pdf]

S1 Table. The most prevalent PIP cases for each set of criteria.

STOPP v. 2		
Section	Drug(s)	Number (%)
Indication of medication	Any drug prescribed without an evidence-based clinical indication	41 (82)
Gastrointestinal System	PPI for uncomplicated peptic ulcer disease or erosive peptic oesophagitis at full therapeutic dosage for > 8 weeks*	16 (32)
Indication of medication	Any duplicate drug class prescription	5 (10)
Drugs that predictably increase the risk of falls in older people	Hypnotic Z-drugs, e.g. zopiclone, zolpidem, zaleplon	4 (8)
Drugs that predictably increase the risk of falls in older people	Neuroleptic drugs	3 (6)
START v.2		
Section	Drug(s)/vaccine(s)	Number (%)
Vaccines	Pneumococcal vaccine at least once after age 65 according to national guidelines	48 (96)
Vaccines	Seasonal trivalent influenza vaccine annually	31 (62)

Musculoskeletal System	Vitamin D and calcium supplement in patients with known osteoporosis and/or previous fragility fracture(s) and/or (Bone Mineral Density T-scores more than -2.5 in multiple sites)	4 (8)
Musculoskeletal System	Bisphosphonates and vitamin D and calcium in patients taking long-term systemic corticosteroid therapy	3 (6)
Analgesics	Laxatives in patients receiving opioids regularly	2 (4)

Amsterdam tool

Section	Drug(s)	Number (%)
Other problems related to the medication status	The indication for the drug is unknown	41 (82)
DRP related to the perspective of the patient	Fear for adverse drug events	10 (20)
Anticoagulant use (use related to heart disease or stroke prevention)	Absence of gastric acid protection in patients ≥ 70 years of age	9 (18)
DRP related to the perspective of the patient	Patient experiences adverse drug event(s)	8 (16)

Artrose and rheumatic diseases	Use of NSAIDs without stomach protection in patients ≥ 65 years of age	7 (14)

Beers 2019

Section	Drug(s)	Number (%)
Drugs to be used with caution: may exacerbate or cause SIADH or hyponatremia	Antipsychotics, carbamazepine, diuretics, mirtazapine, oxcarbazepine, serotonin-norepinephrine reuptake inhibitors, selective serotonin reuptake inhibitors, tricyclic antidepressants, tramadol	26 (52)
PIM: gastrointestinal	Proton-pump inhibitors (>8 weeks unless for high-risk patients)	11 (22)
Drugs to be used with caution: risk of major bleeding	Aspirin for primary prevention of cardiovascular disease and colorectal cancer (adults ≥70 years)	11 (22)
PIM: central nervous system	Nonbenzodiazepine, benzodiazepine receptor agonist hypnotics (ie, “Z-drugs”): eszopiclone, zaleplon, zolpidem	4 (8)
Drugs to be used with caution: risk of GI bleeding	Dabigatran, rivaroxaban (treatment of VTE or atrial fibrillation in adults ≥75 years)	3 (6)

EU(7)-PIM

Section	Drug(s)	Number (%)
Drugs for peptic ulcer and gastro-oesophageal reflux	PPI (>8 weeks) *, e.g. omeprazole, pantoprazole	16 (32)
Other cardiac preparations	Trimetazidine	4 (8)
Anti-dementia drugs	Ginkgo biloba	4 (8)
Antithrombotic agents	Acenocoumarol	3 (6)
Anti-inflammatory and antirheumatic products	Meloxicam	3 (6)

PRISCUS

Section	Drug(s)	Number (%)
Psycholeptic drugs	Zolpidem (> 5 mg/d), lorazepam (> 2 mg/d), alprazolam	6 (12)
Anti-inflammatory and antirheumatic drugs	Meloxicam, ketoprofen	4 (8)
Psychoanaleptic drugs	Fluoxetine, piracetam	4 (8)
Antihypertensive drugs	Doxazosine, reserpine	3 (6)
Antiarrhythmic drugs	Digoxine	1 (2)

* - despite patient’s clinical state and individual indications
