# Supplementary material for: Applicability of tools to identify potentially inappropriate prescribing in elderly during medication review: Comparison of STOPP/START version 2, Beers 2019, EU(7)-PIM list, PRISCUS list, and Amsterdam tool—A pilot study
Source: PLoS One. 2022 Sep 29;17(9):e0275456. doi: 10.1371/journal.pone.0275456 (PMC9521918; doi:10.1371/journal.pone.0275456)
Supplement: S1 Appendix — (PDF) [file pone.0275456.s002.pdf]

S1 Appendix. Interview form.

PRELIMINARY INTERVIEW

Patient's name	
Age/weight/height	
Allergies (including drugs)	
Diet (including beverages: water/grapefruit juice, ml/day)	
Physical activity (how many times a week /how long?)	
Vaccinations (flu, pneumococcus, other)	
Addictions/stimulants: cigarettes (number/day, how many years?)	□ at all, □ 0-1 pack a day, □ >1 pack a day, □ addiction in the past, □ attempt to quit<br>A smoker for (how many?) ………years
alcohol (how many times a week?)	□ at all, □ <2 units a week, □ 2-6 units a week, □ > 6 units a week, □ addiction
coffee (how many cups a day?)	□ at all, □ <2 cups a day, □ 2-6 cups a day, □ > 6 cups a day, □ addiction
Comments on health (including sleep quality)	

Patient's medical history			
Liver disease<br>Kidney disease	Blood pressure	Lipid profile	Glucose

**PATIENT'S DISEASES (SINCE WHEN, PARAMETERS CONTROLLED)**

**LABORATORY TEST RESULTS (DATE, ABNORMALITIES)**

DRUG EXPERIENCE

Description of the patient's drug use behaviour.

□ Requires attention

a. **How does the patient use drugs?**

b. **How does the patient help himself to remember to use drugs?**

MEDICATION REVIEW

Products used by the patient: Rx and OTC drugs, dietary supplements, herbal and homeopathic preparations, vitamins and minerals

**PRODUCT (NAME, STRENGTH, FORM)**	Posology and method of administration	Reason for use	How long?	Does the patient follow the recommendations?	Additional information
