# Supplementary material for: Applicability of tools to identify potentially inappropriate prescribing in elderly during medication review: Comparison of STOPP/START version 2, Beers 2019, EU(7)-PIM list, PRISCUS list, and Amsterdam tool—A pilot study
Source: PLoS One. 2022 Sep 29;17(9):e0275456. doi: 10.1371/journal.pone.0275456 (PMC9521918; doi:10.1371/journal.pone.0275456)
Supplement: S2 Appendix — (PDF) [file pone.0275456.s003.pdf]

S2 Appendix. Data underlying described results.

No.	Age	Gender	Medications (active ingredients)	STOPP/START	Amsterdam	Beers	EU(7)-PIM	PRISCUS
1	65	F	bisoprolol, potassium, pridinol	START: I.1, 2		1.2.		
2	66	F	telmisartan, ursodeoxycholic acid, magnesium, ginkgo biloba, vitamin D3, zolpidem	START: I.2 STOPP: A.1, K.4	100, 116, 123, 124	1.20.	68	N05
3	67	F	indapamide, fluticasone/salmeterol, potassium, magnesium, aspartic acid, zinc, calcium, thiamine, omega-3, lutein, zeaxanthin, selenium, vitamin C, E, copper, glutathione	START: I.1, 2, E.2; STOPP: A.1	116, 123, 124	3.4.		
4	68	F	pantoprazole, valsartan, indapamide, vitamin D3, K2, calcium, red yeast rice, coenzyme Q10, vitamin B6, B12, folic acid, chromium, black radish, artichoke, dehydrocholic acid, peppermint, rutoside, ascorbic acid, denosumab	START: I.1, 2 STOPP: A.1, 3, F.2	56, 115, 116, 123, 124, 84	1.32, 3.4	2	
5	68	F	levothyroxine, metformin, indapamide, amlodipine, allopurinol, magnesium, potassium	START: I. 1, 2; STOPP A.1	116, 122	3.4.		
6	69	F	levothyroxine, pantoprazole, red yeast rice, policosanol, folic acid, co-enzyme Q10, astaxanthin, calcium, vitamin D3, K2, magnesium, bimatoprost, ibuprofen, paracetamol, diclofenac, flax, lemon balm, herbs for indigestion, herbal calming tablets + vit. B6	START: I.2; STOPP: A.1, F.2	116	1.32, 1.34	2, 34, 38	
7	69	F	telmisartan, atorvastatin, cholecalciferol, artichoke extract, ASA, rutoside, ascorbic acid, bamboo, biotin, pantothenic acid	START: I. 1, 2; STOPP: A.1	32, 54, 95, 116, 120, 124			

8	70	F	potassium, torasemide, biotin, rivaroxaban, magnesium, timolol, carvedilol, lercanidipine, rosuvastatin, extract composed of: valerian root, angelica root, lemon balm herb, hop cones, lavender flower; pantoprazole, flax	START: I.1, 2; STOPP: A.1, F.2	116, 124	1.32, 3.4	2	
9	70	F	levothyroxine, calcium, cholecalciferol, trazodone, zolpidem, magnesium + vit. B6, lemon balm, vitamin K2-MK-7, estradiol	START: I.1, 2; STOPP: A.1, K.4	17, 84, 110, 116	1.20, 3.4	59	N05
10	71	F	ASA, bisoprolol, hydrochlorothiazide + telmisartan, rosuvastatin, potassium, white mulberry	START: I.1, 2	46, 54, 56, 124	3.4.		
11	72	F	valsartan, atorvastatin, vitamin D3, K2, milk thistle, rockrose, calcium, magnesium	START: I.2; STOPP: A.1	116, 123, 124, 84			
12	72	F	bisoprolol, pentoxifylline, ASA, nicergoline, simvastatin, perindopril, latanoprost + timolol, vitamin D, risedronate sodium<br>calcium + cholecalciferol, clotrimazole, hydrocortisone, metronidazole	START: I.2; STOPP: A.1, H.8	46, 32, 116	3.1.	21	C04
13	72	F	hydrochlorothiazide + telmisartan, captopril, magnesium + vit. B6, calcium + cholecalciferol, vitamin B12, yeast, diosmin, retinol, tocopheryl, folic acid, glucosamine + lidocaine, naproxen, heparin, meloxicam	START: I.1, 2; STOPP: A.1	54, 116, 123, 124	1.34, 3.4	37	M01
14	73	F	pantoprazole, levothyroxine, allopurinol, potassium, indapamide, ASA, metoprolol, ramipril, latanoprost, glucosamine, nettle leaf, vitamin C, chondroitin, collagen, vitamin C, magnesium	START: I.1, 2; STOPP: A.1, F.2	116	3.1, 3.4	2	

15	74	F	ginkgo biloba, red rice, magnesium, vit. B6, minoxidil, lutein, rosuvastatin, tafluprost, vitamin D, calcium, vitamin K	START: I. 1, 2; STOPP: A.1	116, 120, 122		68	
16	75	F	venlafaxine, indapamide + perindopril, betahistine, Icelandic lichen, ascorbic acid, herbs, choline salicylate, cetalkonium chloride	START: I.1, 2	17	3.4.	66	
17	76	F	levothyroxine, ginkgo biloba, magnesium + vit. B6, Lactobacillus rhamnosus, Lactobacillus helveticus, rosuvastatin, nifuroxazide, drotaverine, calcium, vitamin D and K, diosmin	START: I.1, 2; STOPP: A.1	116, 122, 123		68	
18	76	F	metoprolol, levothyroxine, vitamin B6, B2, lutein	START: I.1, 2; STOPP: A.1	116, 122			
19	76	F	indapamide + perindopril, bisoprolol, ASA, potassium, magnesium, rosuvastatin, estriol	START: D.1; STOPP: H.1, C.2	17, 32, 46, 124	1.24, 3.4, 3.1	28	
20	76	F	nebivolol, perindopril, amlodipine, vitamin K2, cholecalciferol, magnesium + vit. B6, aloe, simvastatin, fenofibrate	START: I.1, 2; STOPP: A.1	116, 120			
21	77	F	pantoprazole, digoxin, bisoprolol, magnesium, potassium, furosemide, iron, allopurinol, furazidine, cholecalciferol, budesonide + formoterol, tiotropium, rivaroxaban	START: I.1, 2, E.2; STOPP: A.1, B.9, F.2	116	1.11, 1.32, 3.4, 4.1	2, 15	C01
22	77	F	nebivolol, amiodarone, valsartan, torasemide, nicardipine, amlodipine, trimetazidine, ASA, omega-3, resveratrol, garlic, magnesium + vit. B6, vitamin D, potassium, pantoprazole, ornithine, choline, collagen, chondroitin, glucosamine	START: I.2; STOPP: A.1, 3, F.2	2, 19, 23, 115, 116, 123	1.13, 3.1, 3.4	2, 16, 17	
23	77	F	venlafaxine, atenolol, furosemide, allopurinol, ASA, atorvastatin, perazine, potassium, magnesium, aspartic acid, zinc, calcium, thiamine, vitamin K2, D3	START: I.2; STOPP: A.1, K.2	46, 54, 116	1.15, 3.4, 3.1	66	

24	77	F	atorvastatin, bisoprolol, lacidipine, potassium, magnesium + vit. B6, trimetazidine, vinpocetine, ASA, vitamin C, calcium + cholecalciferol + menaquinone, senna, iron, selenium, vitamin E	START: I.2; STOPP: A.1	46, 84, 116, 122	3.1.	4, 17	
25	77	F	trazodone, fluoxetine, levothyroxine, sotalol, calcium + vit. D3, cholecalciferol, citicoline, bimatoprost, simvastatin, metamizole, meloxicam	START: I. 2; STOPP: A.1, A.3	32, 54, 15, 115, 116	1.34, 3.4	23, 37 64	C07, M01, N06
26	78	F	perindopril, indapamide, amlodipine, rosuvastatin, ASA, potassium, fluticasone + salmeterol, ipratropium, montelukast, salbutamol, heparin, ascorbic acid + hesperidin + butcher's broom, minerals and vitamins	START: I. 1, 2, E.2, E.3; STOPP A.1	2, 84, 116	3.1, 3.4		
27	78	F	atorvastatin, valsartan, isosorbide, ranitidine, bisoprolol, ASA, nitroglycerin, diclofenac, torasemide, omeprazole, herbs, magnesium, potassium, vitamin B6	STOPP: A.1, A.3, F.2	2, 32, 115, 116	1.32, 3.4, 3.1	1, 2, 34	
28	80	F	glucosamine, chondroitin, calcium, vitamin B6, gliclazide, ramipril, codeine, bilastine	START: I.1, 2; STOPP: A.1	29, 56, 116		70	
29	82	F	levothyroxine, omeprazole, metformin, dabigatran, bisoprolol, magnesium + vit. B6, atorvastatin, vit. K2, D3, calcium, vitamin D	START: I.1, 2; STOPP: A.1, F.2	109, 116	1.32, 3.2	2	
30	83	F	zolpidem, mianserin, quetiapine, omeprazole, clopamide + dihydroergocristine + reserpine, vinpocetine, levocetirizine, bisoprolol, metformin, rosuvastatin, ASA, valsartan, simethicone	START: I. 1, 2; STOPP: A.1, D.10, K. 2, 4, F.2	2, 100, 116, 122	1.8, 1.20, 1.32, 3.4, 3.1, 4.5	2	N05, C02, N02, N04

31	85	F	indapamide, metoprolol, vinpocetine, piracetam, warfarin, mebeverine, lutein, simvastatin, amlodipine, alprazolam, diphenhydramine + paracetamol	START: I.2; STOPP: A.1, D.5, K.1, N1	99, 116	1.1, 1.17, 3.4	55, 67	N05, N06
32	85	F	metformin, acarbose, glimepiride, telmisartan, amlodipine, ASA, atorvastatin, levothyroxine, rutoside, ascorbic acid	START: I.2; STOPP: J.1	46	3.1, 2.1	9	
33	86	F	metoprolol, torasemide, rivaroxaban, calcium, potassium, vitamin D, risedronate, metformin, magnesium, vit. B6, iron, escitalopram, levothyroxine, allopurinol, piracetam, vinpocetine, horse chestnut, ginkgo, hawthorn, mistletoe, arnica	START: I.2; STOPP: A.1	88, 116	3.4, 4.1	67, 68	N06
34	86	F	levothyroxine, hydrochlorothiazide, amlodipine, paracetamol + tramadol	START: I.1, 2, H.2	56	3.4, 4.2	41	
35	88	F	captopril, vitamin K, cholecalciferol, atorvastatin, magnesium + vit. B6, tolterodine, lorazepam, omeprazole, hymecromon, ketoprofen	START: I.1, 2, E.3; STOPP: D.5, K.1, F.2	32, 84, 99, 104, 116	1.17, 1.32, 1.34	2, 31, 53	M01, N05
36	88	F	budesonide + formoterol, ipratropium, silibinin, vinpocetine, dexpanthenol, marigold, vitamin C, omega-3, levothyroxine, verapamil, vitamin D3, sodium, valproic acid<br>valerian, hop cone, passion flower	START: I.2, E.2, E.3; STOPP: A.1	83, 116		26	
37	89	F	bisoprolol, ASA, spironolactone, candesartan, amlodipine, atorvastatin, cilostazol, zolpidem, levothyroxine, meloxicam, paracetamol, drotaverine, herbs, allopurinol, bee putty	START: I.1, 2; STOPP: A.1, K.4	2, 8, 46, 54, 100, 116, 124	1.20, 1.34, 2.1, 3.1, 3.4	37	M01, N05

38	93	F	esomeprazole, tolterodine, chondroitin, apixaban, allopurinol, potassium, vitamin D3, K2, mint, chamomile	START: I.1, 2; STOPP: A.1, F.2	109, 116	1.32.	2, 31	
39	95	F	alkylglycerols, squalene, vitamin D, amlodipine, simvastatin, ASA, nebivolol, furazidine, allopurinol, omega-3, lutein, safflower oil, vitamin C, zinc, mirtazapine, promazine	START: I.1, 2, E.3; STOPP: A.1, K.2	46, 54, 84, 116	1.15, 3.1, 3.4		
40	69	M	metoprolol, valsartan, amlodipine, fenofibrate, atorvastatin, ASA	START: I. 1, 2; STOPP A.1	116			
41	71	M	metoprolol, metformin, enalapril, simvastatin, ASA, iron	START: I.1, 2	46			
42	72	M	simvastatin, ramipril, mianserin, vitamin D, pantoprazole, diclofenac, magnesium, potassium, simethicone, esomeprazole	START: I.1, 2; STOPP: A.1, 3, F.2	115, 116	1.32, 1.34	2, 34	
43	74	M	doxazosin, finasteride, tafluprost, citicoline, collagen, vilcacora, vitamin E, D	START: I.2; STOPP: A.1	116	1.7.	19	C02
44	76	M	amlodipine + indapamide, telmisartan, allopurinol, doxazosin, acenocoumarol, magnesium, vitamin D, vitamin B6, potassium, atorvastatin, simethicone	START: I.2; STOPP: A.1	3, 17, 116	1.7, 3.4	11, 19	C02
45	78	M	valsartan, trimetazidine, vitamin PP, ASA, esomeprazole, clopidogrel, rosuvastatin	START: I.1, 2; STOPP: F.2	10, 123	3.1.	2, 17	
46	80	M	indapamide, simvastatin, amlodipine + ramipril	START: I. 1, 2		3.4.		
47	80	M	bisoprolol, calcium, simvastatin, ramipril, dabigatran, piracetam, omeprazole, acetylcysteine, donepezil, metformin, tamsulosin, finasteride, captopril, paracetamol, drotaverine	START: I.2; STOPP: A.1, D.11, F.2	116	1.32, 3.2	2, 67	N06

48	82	M	loratadine, ramipril, bisoprolol, spironolactone, acenocoumarol, tramadol, potassium, magnesium + vit. B6	START: I.1, 2, H.2; STOPP: A.1, B.12, L.1	116	3.4, 4.2	11	
49	82	M	terpineol, propafenone, rosuvastatin, isosorbide, fenofibrate, lisinopril, bisoprolol, dabigatran, amlodipine, magnesium + vit. B6, pantoprazole, trimetazidine, vinpocetine, saw palmetto, cholecalciferol, lactulose, latanoprost, dorzolamide + timolol, brimonidine	START: I.2; STOPP: A.1, F.2	23, 110, 116	1.32, 3.2	2, 17	
50	83	M	carvedilol, ramipril, torasemide, eplerenone, pantoprazole, acenocoumarol	START: I.2; STOPP: A.1, B.7, B.12, F.2	45, 109 116	1.32, 3.4	2, 11	
